# Draft Genome Sequences of *Bifidobacterium animalis* Consecutively Isolated from Healthy Japanese Individuals

**DOI:** 10.7150/jgen.38516

**Published:** 2020-04-06

**Authors:** Tomoya Tsukimi, Tsubasa Watabe, Kazuki Tanaka, Mitsuhiko P. Sato, Haruo Suzuki, Masaru Tomita, Shinji Fukuda

**Affiliations:** 1 Institute for Advanced Biosciences, Keio University, 246-2 Mizukami, Kakuganji, Tsuruoka, Yamagata 997-0052, Japan.; 2 Systems Biology Program, Graduate School of Media and Governance, Keio University, 5322 Endo, Fujisawa, Kanagawa 252-0882, Japan.; 3 Department of Bacteriology, Faculty of Medical Sciences, Kyushu University, 3-1-1 Maidashi, Higashi, Fukuoka 812-8582, Japan.; 4 Faculty of Environment and Information Studies, Keio University, 5322 Endo, Fujisawa, Kanagawa 252-0882, Japan.; 5 Intestinal Microbiota Project, Kanagawa Institute of Industrial Science and Technology, 3-25-13 Tonomachi, Kawasaki, Kanagawa 210-0821, Japan.; 6 Transborder Medical Research Center, University of Tsukuba, 1-1-1 Tennodai, Tsukuba, Ibaraki 305-8575, Japan.; 7 Metabologenomics, Inc., 246-2 Mizukami, Kakuganji, Tsuruoka, Yamagata 997-0052, Japan.

**Keywords:** Whole-Genome Sequencing, Intestinal Microbiota, *Bifidobacterium animalis*

## Abstract

*Bifidobacterium* species are well recognized as probiotics and colonized in various parts of the human body. Here, we report the draft genome sequences of *Bifidobacterium animalis* isolated from two healthy Japanese volunteers, one of which was sampled twice before and after a 10-year interval. A core genome phylogeny analysis indicated that the strains isolated from the same volunteer were closely related. This paper is the first report of multiple draft genome sequences of *B. animalis* independently isolated from the same individual and provides insight into the probiotic potential of a member of this species.

## Introduction

About 40 trillion of bacteria inhabit the human body, and the majority of these are located in the gastrointestinal tract 1. Previous studies have shown a relationship between the human gut microbiota and various diseases 2,3, and some bacteria, which are referred to as probiotics, are considered to be beneficial for human health 4. The genus *Bifidobacterium* is one well-known group of probiotic bacteria. Species belonging to the genus are gram-positive, rod-shaped, and are anaerobic 5. They colonize in food, such as yogurt, and various parts of the human body including the gut, vagina, oral cavity, and breast milk 5. In the human gut, they ferment carbohydrates into short-chain fatty acids (SCFAs) such as acetate, which have beneficial effects on human health 2. *Bifidobacterium* species do not produce butyrate directly; however, they increase butyrate production via cross-feeding with butyrate-producing bacteria in the colon 6. *Bifidobacterium* species have great potential as a probiotics, but they are not always colonized when they are supplied as a probiotic intervention 7. The genus *Bifidobacterium* contains 69 species and 10 subspecies (http://www.bacterio.net/; accessed 26 February 2019) 8. In this study, we focused on *Bifidobacterium animalis*, which has two subspecies, subsp. *animalis* and subsp. *lactis*. A previous study reported the beneficial effect of the species on ameliorating the risk of food allergy 9 and maintaning remission in ulcerative colitis 10. Here, we report three draft genome sequences of *B. animalis* subsp. *lactis* isolated from two healthy Japanese individuals, one of which was sampled twice before and after a 10-year interval.

## Materials and Methods

### Sample Collection and Isolation of *B. animalis*

Fecal samples were collected from two healthy Japanese volunteers (one subject was 30 years old, and another was 35 and 45 years old at the time of sampling; Table 1) and stored at -80 °C until use.

Each fecal sample (20 mg) was suspended in 1 mL of sterile phosphate-buffered saline (PBS), which was then used to generate two dilutions in PBS, 1:1,000 and 1:10,000. A 50 µL of aliquot from each suspension was inoculated onto a transgalactosylated oligosaccharide (TOS) propionate agar medium plate (Yakult Pharmaceutical Industry Co., Ltd., Tokyo, Japan), which can isolate *Bifidobacterium* from feces 11, and cultured for 1 day at 37 °C under anaerobic conditions. Ten colonies for each subject were randomly selected; inoculated with sterile pipette tips into a 96-well plate (Greiner Bio-One International GmbH, Frickenhausen, Germany) containing 500 µL of De Man, Rogosa and Sharpe (MRS) liquid medium (FUJIFILM Wako Pure Chemical Corporation, Osaka, Japan); and cultured for 1 day at 37 °C under anaerobic conditions.

After cultivation, we confirmed the identity of this species with PCR based on 16S rRNA gene amplification using a *Bifidobacterium*-specific primer 12. Genomic DNA from samples confirmed as *Bifidobacterium* was extracted with the DNeasy Blood & Tissue kit (Qiagen, Hilden, Germany).

### Genome Sequencing, Assembly, and Annotation

The sequencing libraries were prepared using the NEBNext Ultra II FS DNA Library Prep (New England Biolabs, Ipswich, Massachusetts, U.S.) and sequenced with the Illumina HiSeq 2500 (Illumina, Inc. San Diego, California, U.S.). In total 3,282,698 reads (which consisted of 151-bp paired-end reads) were produced (1-Y0; 1,221,120 reads, 2-Y0; 971,770 reads, 2-Y10; 1,089,808 reads). The reads were filtered for read quality using Platanus_trim v1.0.7 (http://platanus.bio.titech.ac.jp/pltanus_trim; accessed 23 February 2019) with default parameters, which resulted in the retention of 3,265,842 reads (1-Y0; 1,215,194 reads, 2-Y0; 965,958 reads, 2-Y10; 1,084,690 reads). Trimmed reads were assembled with Platanus v1.2.4 13 with default parameters, and contigs that were ≤ 300 bp were discarded based on an in-house script deposited in GitHub (https://github.com/MitsuhikoP/cut_short_fasta.git). Genome completeness was estimated using Benchmarking Universal Single-Copy Orthologs (BUSCO) v1 14 with the bacteria data set by submitting assembled sequences (FASTA formatted text file) as query to the gVolante web server 15 and Quality assessment tool for genome assemblies (QUAST) 16. Of the 40 BUSCO group genes, all genes were completely or partially recovered in the assembly and the genomes fraction of three draft genome sequences to complete genome sequence of *B. animalis* subsp. *lactis* DSM 10140 were over 98 % (Table 2). Genes were annotated using the DDBJ Fast Annotation and Submission Tool (DFAST) pipeline 17. Identification of *B. animalis* was performed with BLAST+ v2.4.0 18,19, and pyani v0.2.7 (https://github.com/widdowquinn/pyani; accessed 23 February 2019), which is Python module to calculate average nucleotide identity (ANI). For comparative analysis, complete genome sequences of bacteria in GenBank format of *B. animalis* subsp. *animalis* ATCC 25527 (GCA_000260715.1_ASM26071v1) and subsp. *lactis* DSM 10140 (GCA_000022965.1_ASM2296v1) were downloaded using the command line tool wget from the NCBI FTP site (ftp://ftp.ncbi.nlm.nih.gov/genomes/) on 27 March 2019.

Nucleotide sequence alignments for core genes, which are defined as single-copy genes that are common across all genomes, were produced using Roary v3.12.0 20 with a minimum blastp percentage identity of 95, and MAFFT v7.407 21. A phylogenetic tree was constructed using FastTree v2.1.3 22 with the GTR + CAT model. The phylogenetic tree was rooted with midpoint rooting using the phangorn package v2.4.0 (https://github.com/KlausVigo/phangorn) and drawn using the ape package v5.3 (http://ape-package.ird.fr/) in R v3.4.2 (https://www.r-project.org/). For SNP analysis the nucleotide sequences of 11 genomes of *B. animalis* subsp. *lactis* strain: DSM 10140 (GCA_000022965.1_ASM2296v1), AD011 (GCA_000021425.1_ASM2142v1), B420 (GCA_000277325.1_ASM27732v1), BB-12 (GCA_000025245.1_ASM2524v1), BLC1 (GCA_000224965.2_ASM22496v2), Bi-07 (GCA_000277345.1_ASM27734v1), Bl-04 (GCA_000022705.1_ASM2270v1), Bl12 (GCA_000414215.1_ASM41421v1), CNCM I-2494 (GCA_000220885.1_ASM22088v1), KLDS2.0603 (GCA_000816205.1_ASM81620v1) and V9 (GCA_000092765.1_ASM9276v1) were downloaded using the command line tool wget from the NCBI FTP site on 26 September 2019. The genome alignment of two genomes isolated from volunteer 2 and 11 downloaded genomes and calculating SNPs were performed using MAUVE v20150226 23 with default parameters. Heatmap of SNP matrix was drawn using the corrplot package v0.84 (https://cran.r-project.org/web/packages/corrplot/) in R v3.4.2.

## Results and Discussion

The three draft genome sequences of *B. animalis* analyzed in this study contained 15 contigs consisting of 1,910,072 to 1,918,142 bp, with a G+C content of 60.5% and 1,561 to 1,571 putative coding sequences (Table 2). The complete genome sequence of *B. animalis* is ~1.94 Mbp with a G+C content of 60.5 %, and 1,600 protein-encoding genes 24, which is most consistent with the sequence results of this study.

**Figure 1:**
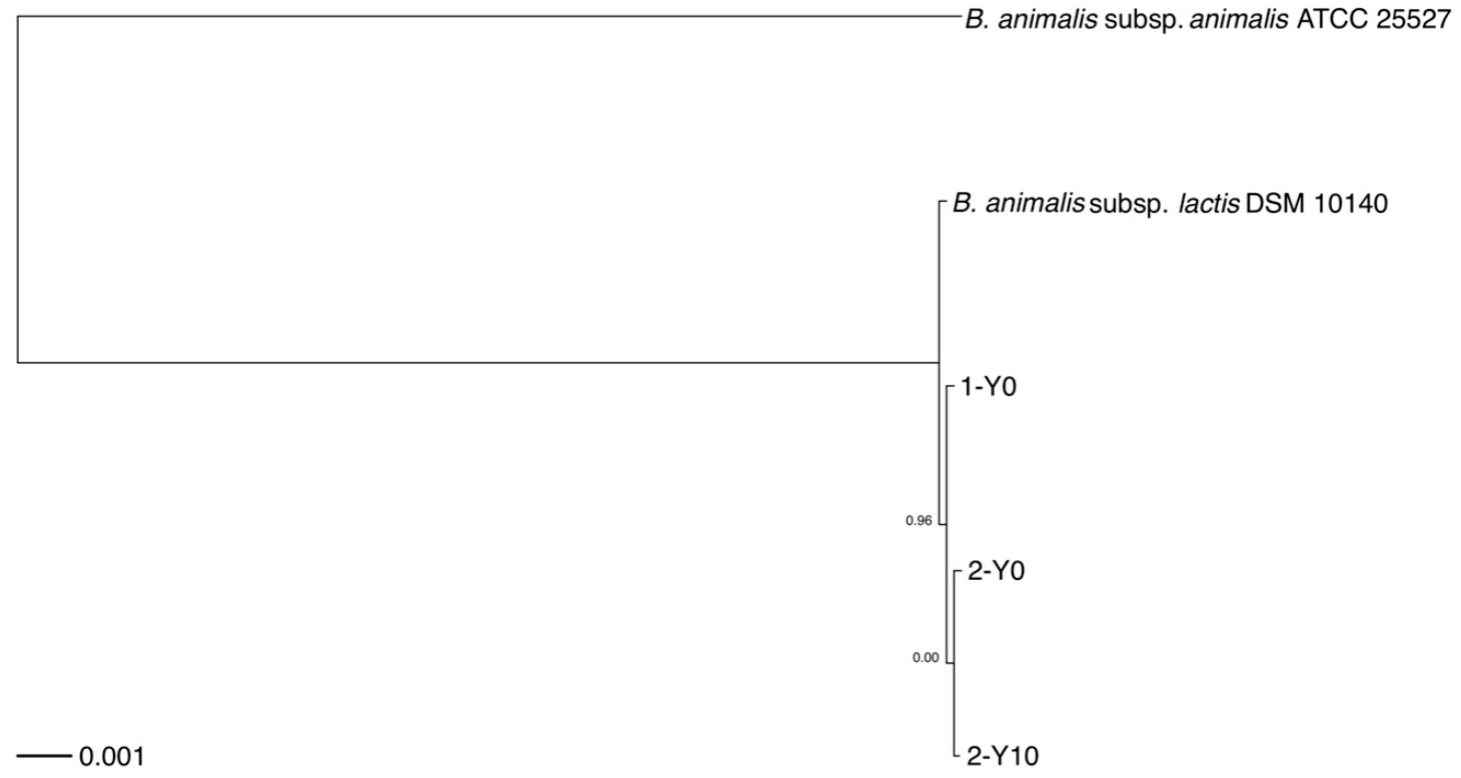
** Rooted phylogenetic tree of strains analyzed in this study.** Rooted phylogenetic tree obtained from a concatenated nucleotide sequence alignment of the 1,176 core genes of five *B. animalis* strains, consisting of the three strains isolated from the two volunteers and reference genomes of two *B. animalis* subspecies. The horizontal bar at the base of the figure represents 0.001 substitutions per nucleotide site. The FastTree branch support values are indicated. 1-Y0, *B. animalis* isolate from volunteer 1; 2-Y0, *B. animalis* isolate from volunteer 2, first collection time point; 2-Y10, *B. animalis* isolate from volunteer 2, second collection time point

To infer the phylogenetic relationships, we built a rooted phylogenetic tree based on a concatenated nucleotide sequence alignment of the 1,176 core genes from 5 strains, included the 3 strains isolated from the volunteers and the two reference genomes of two *B. animalis* subspecies obtained by downloading from NCBI FTP site (Figure 1). The core genome phylogeny indicated that the most basal lineage was *B. animalis* subsp. *animalis*, which was followed by the clade consisting of *B. animalis* subsp. *lactis*, and the strains isolated from the volunteers.

A previous study showed that *B. animalis* subsp. *lactis* was rarely found in intestinal biopsy samples, whereas it was frequently detected in fecal samples 25, indicating that this subspecies detected in human fecal samples may not colonize in the human gut but was supplied as probiotics. However, the volunteer 2 had very similar strains that were sampled at a 10-year interval. This result suggested possibilities that the dietary habits of this volunteer, such as yoghurt intake, had not changed during the 10 years intervention, or that *B. animalis* subsp. *lactis* strains had colonized in his gut.

**Figure 2:**
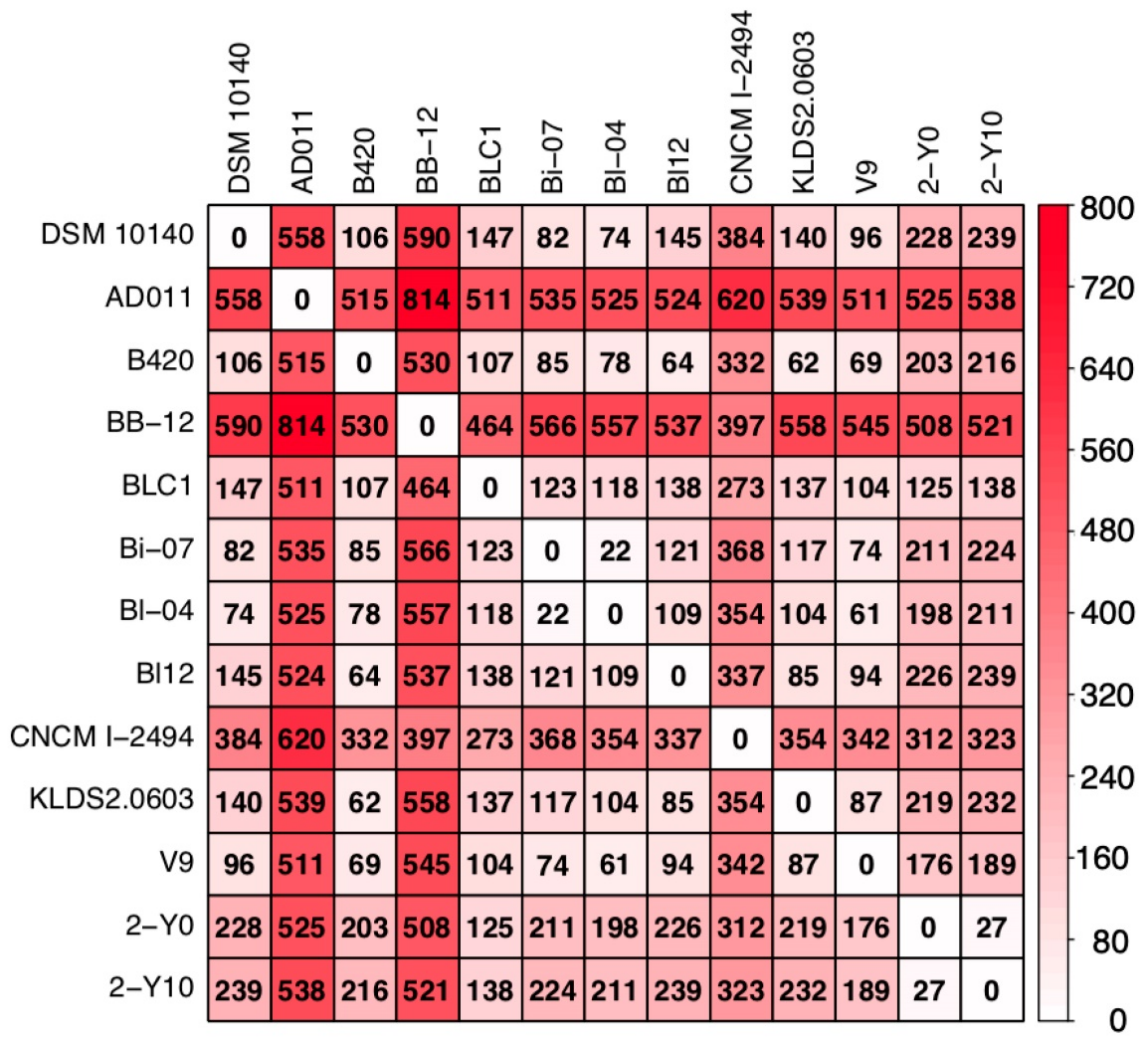
** Heatmap showing the number of single nucleotide polymorphisms (SNPs) in nucleotide sequences of genomes isolated from volunteer 2 and 11 publicly available *B. animalis* subsp. *lactis* strains.** The number in matrix represents the number of putative SNPs between two strains calculated by Mauve. The color represents abundance of SNPs.

To investigate whether strains isolated from volunteer 2 were due to his dietary habits, we compared the number of SNPs between the nucleotide sequences of 11 reference genomes obtained by downloading from NCBI FTP site and that of two genomes isolated from volunteer 2. As the result, the average number of the SNPs between two strains was 281 (the minimum number of SNPs was 22 and the maximum number of that was 814: Figure 2). In Japan, two dairy products containing B. *animalis* subsp. *lactis* are sold: DANONE BIO® (Danone Japan Co., Ltd., Tokyo, Japan), which contains B. *animalis* subsp. *lactis* CNCM I-2494 (https://www.danone.co.jp/bio/about/powerofbe80/be80/; accessed 24 September 2019) and BifiX® (EZAKI GLICO Co., Ltd., Osaka, Japan), which contains B. *animalis* subsp. *lactis* GCL2505 (https://www.glico.co.jp/laboratory/bifix/02.html; accessed 24 September 2019). The number of SNPs between CNCM I-2494 and strains isolated from volunteer 2 (2-Y0; 312, 2-Y10; 323) were more than the average of SNPs (281), which implies that strains isolated from volunteer 2 were not supplied by DANONE BIO® (Figure 2). The genome of B. *animalis* subsp. *lactis* GCL2505 is not registered to NCBI FTP site. Probiotic bacterium supplied by commercial product is probably an identical strain. SNPs between strains isolated from volunteer 2 was 27, which was more than SNPs between Bl-07 and Bi-04. These two strains were differently isolated, and phylogenetically close relationship is consistent with a previous study 26. The result supports that strains isolated from volunteer 2 were not supplied by dietary habits. Detailed survey on dietary habits and a larger number of volunteers are required to conclude whether a strain of *B. animalis* subsp. *lactis* colonize in the human gut.

Previous studies related to the *B. animalis* in the human gut were mainly based on the 16S rRNA amplicon sequence 27-29. This paper is the first report of draft genome sequences of *B. animalis* that includes multiple samples isolated from a single Japanese individual, although the study involved only two volunteers. These results provide insight into the *in vivo* colonization of *Bifidobacterium* and personalized probiotic supplementation.

### Nucleotide sequence accession number

The draft genome sequence of the three strains isolated from the volunteers (1-Y0, 2-Y0, and 2-Y10) has been deposited at GenBank/EMBL/DDBJ under BioProject number PRJDB8215, BioSample number SAMD00168440, SAMD00168441, and SAMD00168442 respectively, and accession number BJKG00000000, BJKH00000000, and BJKI00000000 respectively (accession range: BJKG01000001-BJKG01000015, BJKH01000001-BJKH01000015, and BJKI01000001-BJKI01000015). The version described in this paper is the first version (BJKG01000000, BJKH01000000, and BJKI01000000). The raw reads have been deposited in the DDBJ Sequence Read Archive (DRA) under Submission DRA008418.

## Figures and Tables

**Table 1:** Metadata of subjects in this study.

Subject	Age at time of sampling	Gender	Intake frequency of yogurt	Last intake yogurt before fecal sampling	Strains contained in yogurt
1	30	Male	3 or 4 times a week	Bifidus Yogurt*	*Bifidobacterium longum, Lactobacillus delbrueckii* subsp.* bulgaricus,* *Streptococcus thermophilus*
2	35, 45	Male	a few times a month	not applicable	not applicable

*Bifidus Yogurt is commercial products of MORINAGA MILK INDUSTRY CO., LTD.

**Table 2:** Genomic features of samples used in this study.

Sample	% BUSCO Score (Complete + Partial)	Genome fraction (%)	GC content (%)	Number of contigs	Total contig size (bp)	Largest contig (bp)	N50 (bp)	Number of CDSs
1-Y0	100	98.8	60.5	15	1,917,421	681,743	339,970	1,571
2-Y0	100	98.8	60.5	15	1,918,142	1,021,733	1,021,733	1,561
2-Y10	100	98.4	60.5	15	1,910,072	681,755	339,940	1,561

Sample name represents the subject and the year (Y) after first sampling. GC content (%): The relative frequency (percentage) of guanine and cytosine residues (G + C)/(A + T + G + C) CDSs: coding sequences

## Abbreviations

SCFA: short-chain fatty acid
PBS: phosphate-buffered saline
TOS: transgalactosylated oligosaccharide
MRS: De Man, Rogosa and Sharpe
SNPs: single nucleotide polymorphisms
